# Gunshot Wounds

**Published:** 1881-03

**Authors:** 


### EDITORIAL DEPARTMENT.

" If thou hast truth to utter, speak ;
And leave the rest to God."

GUNSHOT WOUNDS.—" What has been your observation in regard to the appearance of gunshot wounds ? Reply through the journal as early as convenient," And oblige yours, G.

A. Gunshot wounds vary in appearance, necessarily, and for several reasons; such for example, as the size and shape of the projectile, or ball, and the proximity or distance of the victim from the gun or pistol from which the projectile is discharged, and also, as to the part of the body it may penetrate and escape. The muzzel of the gun or pistol, may be so near the wounded person at the time of explosion, that the exposed skin is blackened or burned by the powder, and sometimes, also, even when the ball enters through the apparel, and in all cases, when it enters unexposed portions of the body, at either short or long range, particles of clothing are likely to be driven into the wound by it. A leaden projectile entering and passing through a portion of the body, without encountering bone, in its transit, will produce certain marks, which will enable one to decide with much certainty the points of entrance and exit, e. g.: At the entrance the skin will often be turned into the wound, around its margin, and it is usually round or oblong in shape, and almost always smaller than the exit wound. The latter is not only the larger, but usually more lacerated, and likely to be elevated above, than depressed below the surface, as is often the case at the point of entrance.

When a ball encounters bone, or cartilage in its transit, and becomes flattened or otherwise changed in shape, the external wound is rendered larger than otherwise, and when a fragment of bone, or cartilage is driven out with it, the solution of continuity is always further increased in size. For several reasons, which will readily suggest themselves to the surgeon, and need not, therefore, be mentioned here, it is often matter of great importance to determine the points of entrance and exit of a projectile ! In some cases medico-legal questions of the greatest moment, arise on the entrance or exit of a ball or shot; and a knowledge of the facts just stated, may avail much in deciding an important matter.

As already alluded to, there are other, and what might be called collateral facts, in connection with the appearance of wounds produced by the entrance and exit of leaden balls, or projectiles; but what has been said will be sufficient to guide any one in forming a proper diagnosis in a given case.

